# Anti-*Leishmania major* activity of *Calotropis procera* extract by increasing ROS production and upregulating TNF-α, IFN-γ and iNOS mRNA expression under in vitro conditions

**DOI:** 10.1186/s41182-024-00578-4

**Published:** 2024-02-01

**Authors:** Shahla Amani, Soheila Alinejad, Negar Asadi, Elham Yousefi, Shahram Khademvatan, Gordon Stanley Howarth

**Affiliations:** 1 Cellular and Molecular Research Center, Cellular and Molecular Medicine Institute & Department of Medical Parasitology and Mycology, Urmia University of Medical Sciences, Urmia, Iran; 2 School of Animal and Veterinary Sciences, The University of Adelaide, Roseworthy, South Australia Australia

**Keywords:** Amastigote, *Calotropis procera*, *Leishmania major*, Promastigote, Gene expression, PBMCs

## Abstract

**Background:**

Leishmaniasis, caused by protozoan parasites of the genus *Leishmania*, is a neglected tropical disease with 700,000 to 1,000,000 global new cases annually. Adverse effects associated with expense, long-term treatment and drug resistance have made conventional therapies unfavorable, encouraging the search for alternative drugs based on plant products. In this study, the effect of *Calotropis procera* (Asclepiadaceae) extract against viability of promastigotes and amastigotes of *Leishmania major* was evaluated in vitro.

**Methods:**

The extract from the leaves of *C. procera* seedlings was prepared using a methanol maceration method. The colorimetric cell viability 3-(4,5-dimethylthiazol-2-yl)-2,5-diphenyltetrazolium bromide (MTT) assay was used to determine the growth-inhibitory effect of the extract on promastigotes. The level of reactive oxygen species (ROS) in promastigote cultures was determined after treatment with the extract using the 2',7'-dichlorofluorescein diacetate (DCFH-DA) method and compared with untreated cultures (control). After exposure to the extract the expression levels of tumor necrosis factor-α (*TNF-α*), interferon gamma (*IFN-γ*) and inducible nitric oxide synthase (*iNOS*) genes were determined and compared to control in peripheral blood mononuclear cells (PBMCs) infected with *L. major*.

**Results:**

Based on the MTT assay, the *C. procera* extract significantly reduced the proliferation of *L. major* promastigotes with IC_50_ values of 377.28 and 222.44 μg/mL for 24 and 72 h, respectively (*p* < 0.01). After treatment with 222.44 and 377.28 μg/mL of *C. procera* extract, ROS production in *L. major* promastigote cultures increased 1.2- to 1.65-fold and 2- to 4-fold compared to the control, respectively (*p* < 0.05). *C. procera* extract induced significant increases in gene expression of *TNF-α* (2.76–14.83 fold), *IFN-γ* (25.63–threefold) and *iNOS* (16.32–3.97 fold) in infected PBMCs compared to control (*p* < 0.01).

**Conclusions:**

On the basis of its anti-leishmanial activity, *C. procera* can be considered as a promising new plant source for the potential treatment of leishmaniasis.

## Background

Leishmaniasis is a neglected obligatory intracellular tropical disease, caused by different species of *Leishmania* parasites, that is prevalent in many parts of the world [1]. Leishmaniasis is transmitted by the bites of infected female sand flies [2]. These parasitic protozoans have two distinct stages in their life cycle: promastigotes (extracellular flagellated promastigotes in the gut of the female sand fly vector which can be injected into the host dermis by vector bite), and amastigotes (transformation of promastigotes into intracellular amastigotes after internalization by host phagocytotic cells through phagocytosis) [3, 4]. At the vector bite site, the parasite in promastigote form attacks host phagocytotic cells (inflammatory monocytes, macrophages and neutrophils) and then transform into intracellular amastigotes [4, 5]. Amastigotes are able to proliferate within monocytes/macrophages and transmit the infection to other macrophages, neutrophils, monocytes, some dendritic cells and fibroblasts [4, 6, 7].

The defense reactions of host cells against *Leishmania* infection are based on the co-ordination of two host immune systems, innate immunity (complement-mediated lysis) and adaptive immunity (Th1-mediated response) [4, 5]. The expression of activating cytokines such as IFN-γ and TNF-α is essential for parasite proliferation control [8]. When activated by cytokines, host cells can suppress the infection by killing intracellular parasites [9]. The production of reactive oxygen species (ROS) and Nitric Oxide (NO expressed by inducible nitric oxide synthase (iNOS) gene) represent as two major effective leishmanicidal molecules for exclusion of intracellular parasites without damaging the host cell [4, 5].

Treatment of leishmaniasis has always been challenging. The absence of effective immunizations and/or emergence of treatment resistance have all contributed to the rise in prevalence of this disease [10]. Pentavalent antimonials, amphotericin B, paromomycin and pentamidine are the most regularly used drugs for leishmaniasis therapy. However, they have significant side effects, require high dosages for extended periods of time, and are supplied parenterally [11]. An effective and economical new treatment approach would be advantageous in overcoming the difficulties induced by leishmaniasis chemotherapy. In the treatment of parasitic infections, phytotherapy has recently attracted attention as a viable alternative to chemotherapy [12]. In this regard, plant studies have been expanded to discover new secondary metabolites with increased bioactivity and fewer side effects [12, 13].

*Calotropis procera* (Asclepiadaceae) is a common plant throughout the world (growing mainly in dry and semi-arid climates), renowned for its conventional therapeutic uses including the treatment of infectious diseases, skin and dermal illness (infections, leprosy, wounds, psoriasis), respiratory diseases (bronchial asthma and cough), gastrointestinal diseases (dysentery, constipation and nematode infections), urinary tract diseases (kidney stones and chronic renal problems), jaundice, malaria, fever, earache, neuropsychiatric disorders, liver diseases and even tumors [14–19]. Recently, *C. procera* extracts have been reported to exert anticancer, anti-inflammatory, antidiabetic, gastroprotective, cardiovascular, antipyretic, antioxidant, antimalarial, anthelmintic, antifungal, anti-angiogenic, hypolipidemic, antibacterial, analgesic and anticonvulsant properties [14, 18, 20–25]. *C. procera* leaf extract has also demonstrated effective anti-leishmanial activity against promastigotes of *L. tropica*, mediated via a mechanism of apoptosis induction [26].

The mechanisms and pathways that stimulate *leishmania*-infected macrophages are of special interest since they hold potential for the development of new treatment and prevention strategies. In the present research, we sought to evaluate the effect of *C. procera* extract on PBMCs infected with *L. major* and the expression levels of *INF-γ*, *TNF-α* and *iNOS* genes. It was hypothesized that treatment of *L. major* promastigotes and amastigotes with *C. procera* extract would induce ROS production and upregulation of *INF-γ*, *TNF-α* and *iNOS* genes which could be effective for parasite control.

## Methods

### Preparation of plant extract

**Fig. 1:**
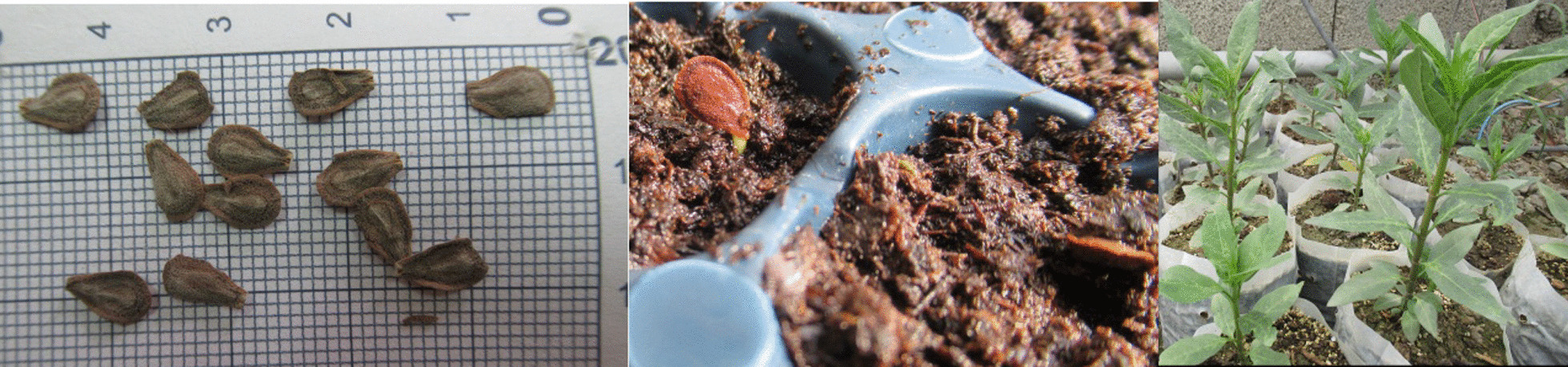
Seeds and seedlings of *Calotropis procera*

The seeds of *C. procera* were provided and cultivated in the greenhouse (Fig. 1) by Zarringiah Co., West Azerbaijan Province, Urmia, Iran. The growing seedlings were authenticated by a taxonomist. Voucher specimens (voucher numbers: CP/1397 433) were deposited in the Zarringiah Co. Herbarium, Urmia, Iran. The leaves of 6 weeks seedlings were carefully harvested for extraction (Fig. 1). After washing and shade drying at room temperature, the samples (500 mg) were powdered and extracted by 10 ml 80% methanol (Merck, Darmstadt, Germany) maceration method and shaking incubation at 25 ± 2  C for 72 h. The extract was paper filtered and the residue re-macerated for the second (80% methanol, 48 h) and third (80% methanol, 24 h) time. Finally, the solvent was evaporated in a vacuum rotary evaporator (Rotary Evaporator N-1110, Eyela, Tokyo, Japan). The concentrated residue was frozen at − 20  C. The dried powders were dissolved in phosphate-buffered saline (PBS, Cl_2_H_3_K_2_Na_3_O_8_P_2_, 1X, pH 7.4, Gibco, Paisley, UK) and diluted to prepare test concentrations of extract.

### Cultivation of *Leishmania major* parasite

The Iranian standard reference strain of *L. major* promastigotes (MRHO/IR/75/ER) was provided by the Department of Medical Parasitology and Mycology, Urmia University of Medical Sciences, Urmia, Iran. The promastigotes were cultured in RPMI-1640 culture medium (+ HEPES and L-glutamine, Gibco, Paisley, UK) supplemented with 10% (v/v) heat-inactivated fetal bovine serum (FBS, PAN Biotech, Aidenbach, Germany) and antibiotics (100 units/ml penicillin, and 100 μg/ml streptomycin, Sigma-Aldrich, St. Louis, Missouri, USA). The cultures were placed in an incubator shaker (120 rpm) at 25 ± 1  C and grown until reaching the stationary growth phase.

### MTT viability assay

In order to determine the growth-inhibitory effect of *C. procera* extract, the promastigotes of *L. major* (1 × 10^6^ parasites/mL) at stationary phase were added (triplicate) to the 96-well plate and treated with extract concentration range of 0–400 µg/mL. Wells containing culture medium without promastigote as blank sample, glucantime-treated promastigote (Glucantime®: Sanofi, France) as positive control and PBS-treated promastigote as negative control were used. The plates were incubated for 24 and 72 h at 25 ± 1  C. Tetrazolium salt 3-(4,5-dimethylthiazol-2-yl)-2, 5-diphenyltetrazolium bromide (MTT, Sigma-Aldrich, St. Louis, Missouri, USA) was dissolved in PBS (Gibco, Paisley, UK) at 5 mg/mL, added to each well (10% of the volume of well). After incubation at 25 ± 1  C for 4 h and adding dimethyl sulfoxide (DMSO, Sigma-Aldrich, St Louis, Missouri, USA) to stop the reaction, the absorbance was read by plate reader (Stat Fax 2100 ELISA Plate Reader, Awareness Technology, Palm City, Florida, USA) at 545–600 nm:

$$\% {\text{ cell viability}}\, = \,\left( {{\text{absorbance of treated wells}}{-}{\text{blank}}} \right)/{\text{ absorbance of control}}{-}{\text{blank}})\, \times \,{1}00.$$

The logarithmic regression analysis of dose–response curve was used for calculation of 50% inhibitory concentration of extract (IC_50_) and 50% cytotoxic concentration of extract (CC_50_) using GraphPad Prism 5.0.4 software (GraphPad Software, San Diego, California, USA).

### Reactive oxygen species (ROS) levels

ROS production was detected using the fluorescent 2,7-dichlorodihydrofluorescein diacetate (H2DCFDA) dye according to the kit instructions (ROS assay kit: KROS96, Kiazist Life Sciences, Iran), as described by Mendoca et al. [27]. After treatment with or without extract at 100 µg/mL (< IC_50_), 222.44 µg/mL (IC_50_-72 h) and 377.28 µg/mL (IC_50_-24 h) concentrations for 3, 6, and 12 h, *L. major* promastigotes were washed with ROS buffer and incubated with DCFDA reagent for 45 min in darkness. The fluorescence intensity was immediately measured as EX / EM = 485/535 nm and analyzed by flow cytometry (PAS Particle Analysing System, Partec, Germany). Glucantime (10 μg/mL) and PBS (Gibco, Paisley, UK) were used as positive and negative controls, respectively.

### Cultivation of PBMCs and infection with *L. major* promastigotes

Peripheral blood mononuclear cells were isolated from healthy heparinized blood as described by Srivastava et al. [28] and cultured in 6-well plates (10^5^ cells/well) containing RPMI 1640 medium (+ HEPES and L-glutamine, Gibco, Paisley, UK, 10% FBS (PAN Biotech, Aidenbach, Germany), 100 U/ml penicillin–100 µg/mL streptomycin (1% P/S, Sigma-Aldrich, Missouri, USA) as antibiotics) at 37  C – 5% CO_2_. The adherent cells were infected with *L. major* promastigotes at stationary growth phase (10:1 parasites/cell) for 4 h and washed three times with PBS (Gibco, Paisley, UK) to remove free parasites. After stabilization of infected cells (amastigote-containing cells) the treatments were performed for 24, 48 and 72 h including negative control, 377.28 μg/mL *C. procera* leaf extract (24 h-IC_50_ concentration) and 222.44 μg/mL *C. procera* leaf extract (72 h-IC_50_ concentration).

To determine the cytotoxicity effect of *C. procera* leaf extract on PBMCs, the concentration of extract required to reduce uninfected PBMC growth by 50% after 24–72 h was calculated using the MTT assay.

### IFN-γ, TNF-α and iNOS mRNA determination by real-time PCR

Total RNA from infected PBMCs (treated or untreated with extract) was extracted using SinaClon RNXplus kit (SinaClon, Tehran, Iran). Synthesis of cDNA was performed with 1 μg of total RNA using the AccuPower® CycleScript RT PreMix Kit (Bioneer, Daejeon, South Korea) according to the manufacturer’s instructions. The specific primers targeting the genes were designed as listed in Table 1, and manufactured (Nedaye Fan Co, Tehran, Iran). The Real-time RT-PCR assays were performed by SYBR Green detection (SYBR Green qPCR Master Mix, Thermo Scientific/Fermentas, Vilnius, Lithuania) and the relative quantification (2^−ΔΔCT^ method) was applied, using the homo-sapiens β-actin gene as the reference control. Real-time RT-PCR reactions were conducted using three-step real-time MicPCR (Bio Molecular system, Upper Coomera, Queensland, Australia) in 20 μL total volume containing 10 μL SYBR Green Master Mix, 2 μL of 1:20 diluted cDNA (50 ng), 0.5 µL of each primer (10 µM), and 7 µL nuclease-free water. The real-time PCR temperature program consisted of a hold at 95˚C for 10 min followed by 40 thermal cycles of 95˚C for 15 s, primer annealing temperature (Table 1) for 20 s, and 72  C for 30 s.

**Table 1:** The specific primers for *β-actin*, cytokine (*IFN-γ* and *TNF-α*) and *iNOS* genes

Primer name	Sequence (5' to 3')	Base/length	TA (˚C)	Product size (bp)
β-Actin—forward	TGCCGACAGGATGCAGAAG	19	58	106
β-Actin—reverse	GCCGATCCACACGGAGTACT	20		
IFN-γ- forward	AGCTCTGCATCGTTTTGGGTT	21	54	118
IFN-γ- reverse	GTTCCATTATCCGCTACATCTGAA	24		
TNF-α-forward	CACGCTCTTCTGCCTGCTG	19	56	105
TNF-α-reverse	GATGATCTGACTGCCTGGGC	20		
iNOS- forward	ATAGAGGATGAGCTGAGCATTCCA	24	58	109
iNOS- reverse	GCGTTACTCCACCAACAATGGCAA	24		

### Statistical analysis

Values were expressed as the mean of triplicate samples ± standard deviation (SD). The results were analyzed statistically by one-way ANOVA test followed by Duncan’s multiple range tests (*p* < 0.05).

## Results

### In vitro leishmanicidal activity of *C. procera* against *L. major* promastigotes

**Fig. 2:**
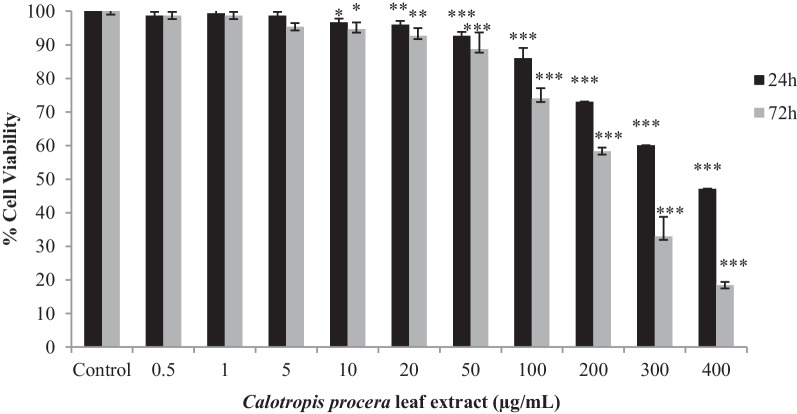
MTT assay of *L. major* promastigotes viability after *C. procera* leaf extract treatments. Data are expressed as mean of % cell viability ± standard deviation. Significant statistical differences in relation to control are indicated as (*), (**), and (***) at the 0.05, 0.01 and 0.001 levels, respectively

The effect of *C. procera* leaf extract on *L. major* promastigotes was monitored after 24 and 72 h of treatment. The extract showed a dose-dependent reduction in promastigote proliferation (Fig. 2), with 50% growth inhibition of the promastigotes at 377.28 μg/mL extract after 24 h and 222.44 μg/mL extract after 72 h of treatment. In order to ensure the selectivity of *C. procera* leaf extract to act only against intracellular amastigotes, the cytotoxicity against PBMCs was investigated. No cytotoxicity was observed at the concentrations analyzed (selectivity index (SI) higher than 4, Table 2).

**Table 2:** In vitro activity of *C. procera* against *L. major* promastigotes and its cytotoxicity for PBMCs

Treatments	IC_50_ (µg/mL)	CC_50_ (µg/mL)	SI
*C. procera* leaf extract			
24 h	377.28 ± 0.16	1576.67 ± 25.17	5.28
72 h	222.44 ± 18.87	1164.33 ± 11.24	4.33
Glucantime			
24 h	14.02 ± 0.13	Nd	–
72 h	5.5 ± 0. 11	Nd	–

IC_50_: inhibitory concentration of *C. procera* leaf extract and glucantime for 50% of the promastigotes

CC_50_: cytotoxic concentration of *C. procera* leaf extract for 50% of cells

SI: selectivity index = CC_50_ PBMCs/IC_50_ promastigote, SI > 1.0: not toxic for normal cells

nd: not determined

### *C. procera* extract increased ROS production

**Fig. 3:**
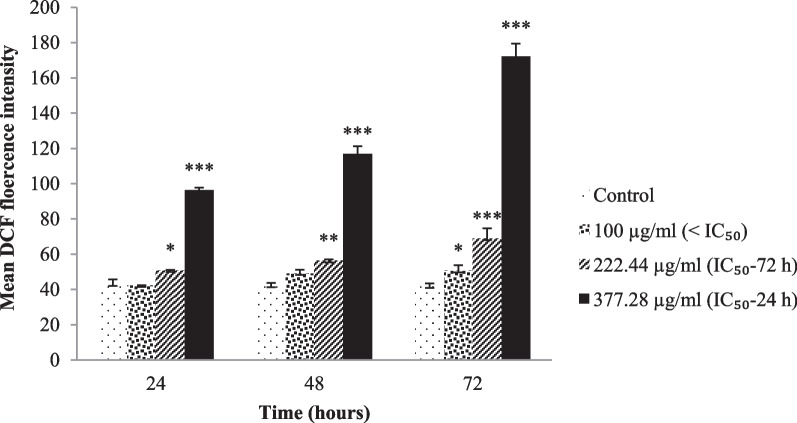
ROS production in *C. procera* leaf extract- treated *L. major*. Promastigotes were treated with 100 ( < IC_50_), 222.44 (IC_50_-72 h) and 377.28 (IC_50_-24 h) µg/mL for 24, 48 and 72 h at 25 ± 1  C. Cells (control and treated cultures) were incubated with probe H2DCFDA (green fluorescent dye). Intracellular ROS production was analyzed by flow cytometry. Data are expressed as mean of DCF fluorescence intensity ± standard deviation. Significant statistical differences in relation to control are indicated as (*), (**), and (***) at the 0.05, 0.01 and 0.001 levels, respectively

Overproduction of ROS in mitochondria is one of the important defense weapons of the cell against pathological and physiological threats that lead to oxidative stress. We determined the ROS production in *L. major* promastigotes using fluorescent H2DCFDA detection by flow cytometry. Promastigotes treated with 222.44 and 377.28 µg/mL *C. procera* extract significantly enhanced ROS production by 1.65 and 4 times (*p* < 0.001), respectively, compared to controls (Fig. 3).

### *C. procera* extract increased expression of IFN-γ and TNF-α transcripts

**Fig. 4:**
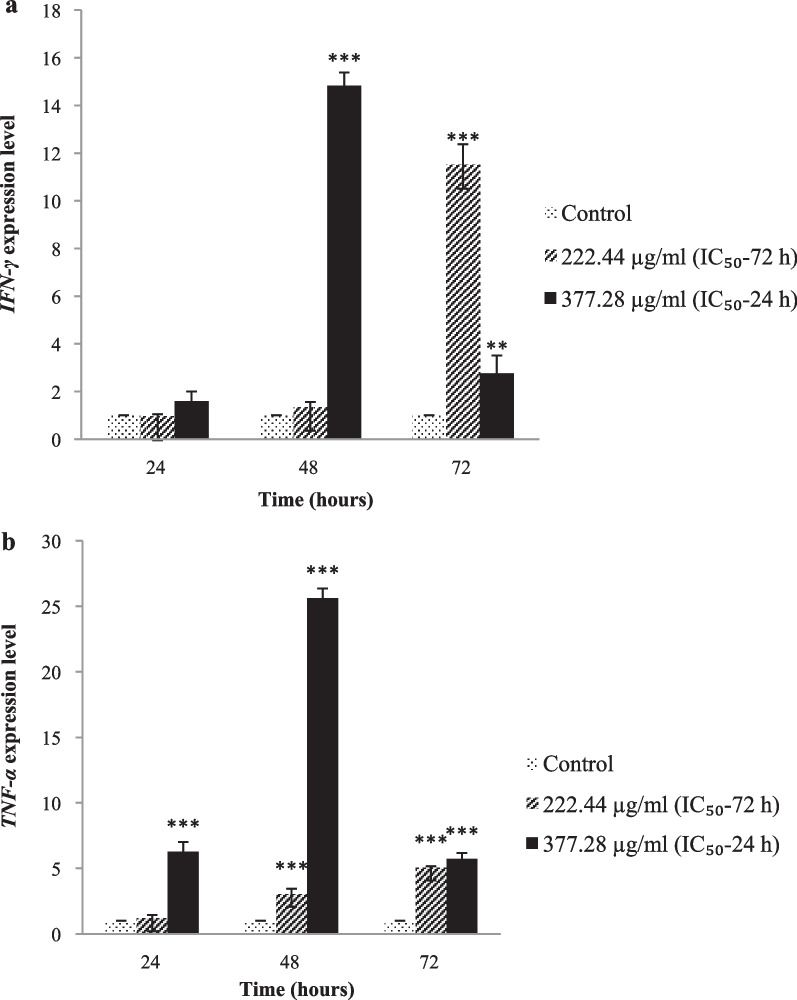
Expression level of *IFN-γ* (**a**), *TNF-α* (**b**) in *L. major*-infected PBMCs treated with *C. procera*. The highest levels of *IFN-γ* and *TNF-α* expression were detected at 48-h treatment with 377.28 µg/mL *C. procera* extract. Significant statistical differences in relation to control are indicated as (*), (**), and (***) at the 0.05, 0.01 and 0.001 levels, respectively

As shown in Fig. 4, in *L. major*-infected PBMCs, IFN-γ and TNF-α mRNA expression increased significantly during treatment with *C. procera,* depending on exposure time (*p* < 0.01). The highest expression of both *IFN-γ* and *TNF-α* genes was detected at 48 h treatment with 377.28 µg/mL *C. procera* extract, compared to control expression levels (*p* < 0.001). In the presence of 222.44 µg/mL *C. procera* extract, cytokine expression in *L. major*-infected PBMCs significantly increased with increasing exposure time (*p* < 0.001), such that 72 h treatment induced higher levels of *TNF-α* and *IFN-γ* in comparison with control, respectively (Fig. 4).

### *C. procera* increased iNOS mRNA expression

**Fig. 5:**
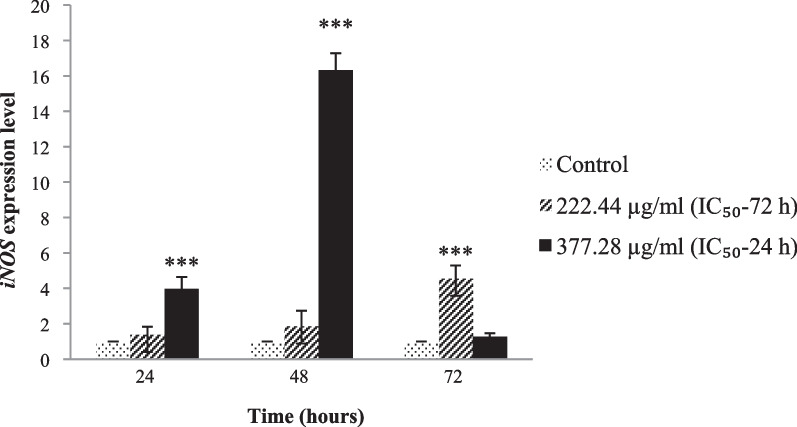
Expression level of *iNOS* transcripts in *L. major*-infected PBMCs treated with *C. procera* leaf extract. The highest level of *iNOS* expression was detected at 48-h treatment with 377.28 µg/mL *C. procera* extract. Significant statistical differences in relation to control are indicated as (*), (**), and (***) at the 0.05, 0.01 and 0.001 levels, respectively

As hypothesized, the level of iNOS mRNA expression was significantly increased (4–16-fold, *p* < 0.001) by *C. procera* extract in *L. major*-infected PBMCs compared to control (Fig. 5). In *L. major*-infected PBMCs, induction of *iNOS* expression was significantly increased 4.59-fold after 72-h treatment with 222.44 μg/mL and 3.97- to 16.32-fold following 24- to 48-h treatment with 377.28 µg/mL (*p* < 0.001, Fig. 5).

## Discussion

The current study represents the first report of *C. procera* and its impact on the expression of relevant genes in *L. major*-infected PBMCs. Our findings demonstrated that *C. procera* could induce ROS generation in *L. major* promastigotes; increase expression of *IFN-γ* and *TNF-α* cytokine genes, together with nitric oxide synthase expression, in *L. major*-infected PBMCs. Considered together, this indicated an inhibitory effect on the proliferation of *L. major* promastigotes.

*C. procera* is known to possess antioxidant, antipyretic, antifungal, antimicrobial, analgesic, anti-inflammatory and antinociceptive properties which have been attributed to its phytochemical composition [18, 22–24]. In the current study, the anti-leishmanial effects of *C. procera* on *L. major* promastigotes were evaluated by MTT assay. Our findings showed that *C. procera* had a dose-dependent cytotoxic effect against *L. major* promastigotes, as also reported against *L. tropica* species [26]. The IC_50_ value obtained from leaf extract of *C. procera* against *L. major* promastigotes in the present analysis was 377.28 μg/mL at 24-h treatment and 222.46 μg/m at 72-h treatment which was higher than the value reported (66.8 μg/mL -72 h) in Al Nasr' study [29]. It has been clear that the biosynthesis and accumulation of secondary metabolites in plants are influenced by genetic and environmental factors [30]. Therefore, it is not far from expected that the IC_50_s of the same plant but grown in different environmental conditions are different. *C. procera* comprises secondary metabolites such as phenolic compounds, flavonoids, cardiac glycosides, terpenoids, saponins and sterols [17]. The anti-leishmanial effects of these phytochemicals against *Leishmania* spp. have been demonstrated previously [31]. Therefore, these phytochemicals may have been responsible for the anti-leishmanial activity of *C. procera* in the current study. Phenolic acids such as gallic acid and ellagic acid [32], and flavonoids, for example, rutin [33] have shown growth-inhibitory effect against promastigotes and amastigotes of *L. major* and *L. donovani*, respectively.

Based our results, in a dose–time-dependent manner, *L. major* promastigote proliferation was inhibited after treatment with *C. procera* extract as a consequence of increased ROS production. It can be stated that after mitochondrial dysfunction and as a result leakage in the electron transport chain, the level of ROS in Leishmania promastigotes will exceed its basal level [34]. Phytochemicals possessing anti-leishmanial activity may therefore have been able to increase ROS levels, resulting in oxidative stress produced by ROS, thereby causing cell death [31]. Similarly, other studies have pointed out the important role of herbal products in inducing excessive production of ROS and subsequent cell death of *Leishmania* spp. For example, Dehydroabietic acid isolated from *Pinus elliottii* in the Gonçalves, et al. study [35] and total phenolic fraction from extra virgin olive oil in the Karampetsou, et al. study [34], promoted cellular ROS production in *L. amazonensis* and *L. major* parasites, respectively.

In the current study, 48 h treatment with 377.28 µg/mL *C. procera* induced *IFN-γ* and *TNF-α* expression and increased *iNOS* gene expression in *L. major-*infected macrophages, leading to elimination of the parasites. To success of host's defense mechanisms against *Leishmania* parasite, IFN-γ and TNF-αas pro-inflammatory cytokines play crucial role [36]. Evidently, FN-γ and TNF-α have synergistic effects in killing of *Leishmania major* by stimulating macrophages to increase ROS and reactive nitrogen species (RNS) production [4, 5, 36]. As a result of increased *iNOS* gene expression, host cells produce NO via activity of the iNOS enzyme [37]. NO is known to be a major leishmanicidal agent, such that deficiency or inhibition of NO production leads to parasite resistance or survival, respectively [37]. It has been demonstrated that injection of L-NG-monomethyl arginine (L-NMMA), as a NO inhibitor, into the lesions in *L. major*-infected CBA mice caused disease exacerbation by 10(4)-fold increasing in the number of parasites [38].

According to these findings, increased *iNOS* expression could represent an effective mechanism for *C. procera* to control *Leishmania* infection; supported by the RT-PCR results in the current study. It is evident that antimonials, amphotericin B and other anti-leishmanial agents have been shown to combat parasites by increasing production of ROS and NO [39, 40]. Similarly, the increased release of IFN-γ and TNF-α by *C. procera* leaf extract could therefore represent an underlying immune mechanism to stimulate *iNOS* expression and thus NO production. Considering the safety, accessibility and low cost of bioactive compounds, medicinal extracts such as *C. procera* can be a valuable natural source of anti-leishmanial agents.

## Conclusions

In the current study, *C. procera* leaves extract exerted significant anti-leishmanial activity against *L. major* promastigotes and amastigotes. This was likely mediated by effective concentrations of *C. procera* extract increasing the levels of ROS in promastigotes, and increasing the expression levels of *IFN-γ*, *TNF-α* and *iNOS* genes in PBMCs containing amastigotes. Considering that despite the high efficacy rate, the presence of severe side effects, toxicity, some drug resistance and the high cost of chemical anti-leishmanial drugs encourage efforts to find effective, safe and cost-effective natural agents against *Leishmania*, *C. procera* can be considered as a new source of natural anti-leishmanial agents. Certainly, to better understand the effect of *C. procera* leaf extract on leishmaniasis infection, its active and non-toxic components should be studied further utilizing an experimental murine model.

## Data Availability

All data generated or analyzed during this study are included in the manuscript.

## Abbreviations

CC_50_: 50% Cytotoxic concentration
DCFH-DA: 2',7'-Dichlorofluorescein diacetate
DMSO: Dimethyl sulfoxide
FBS: Fetal bovine serum
IC_50_: 50% Inhibitory concentration
IFN-γ: Interferon gamma
iNOS: Inducible nitric oxide synthase
MTT: 3-(4,5-Dimethylthiazol-2-yl)-2, 5-diphenyltetrazolium bromide
NO: Nitric oxide
PBMCs: Peripheral blood mononuclear cells
PBS: Phosphate-buffered saline
ROS: Reactive oxygen species
SD: Standard deviation
SI: Selectivity index
TNF-α: Tumor necrosis factor-α
